# Role of Innate Immunity in the Pathogenesis of Chronic Rhinosinusitis: Progress and New Avenues

**DOI:** 10.1007/s11882-012-0249-4

**Published:** 2012-02-05

**Authors:** Cornelis M. van Drunen, Jenny M. Mjösberg, Christine L. Segboer, Marjolein E. Cornet, Wytske J. Fokkens

**Affiliations:** 1 Department of Otorhinolaryngology, Academic Medical Center, Amsterdam, The Netherlands; 2 Tytgat Institute for Liver and Intestinal Research, University of Amsterdam, Amsterdam, The Netherlands

**Keywords:** Innate immunity, Chronic rhinosinusitis, Pathogenesis, Nasal polyposis, Pattern-recognition receptors, Epithelium, ILC2, Microorganisms

## Abstract

Chronic rhinosinusitis is a heterogeneous and multifactorial disease with unknown etiology. Aberrant responses to microorganisms have been suggested to play a role in the pathophysiology of the disease. Research has focused on the presence, detection, response to, and eradication of these potential threats. Main topics seem to center on the contribution of structural cells such as epithelium and fibroblasts, on the consequences of activation of pattern-recognition receptors, and on the role of antimicrobial agents. This research should be viewed not only in the light of a comparison between healthy and diseased individuals, but also in a comparison between patients who do or do not respond to treatment. New players that could play a role in the pathophysiology seem to surface at regular intervals, adding to our understanding (and the complexity) of the disease and opening new avenues that may help fight this incapacitating disease.

## Introduction

Research into the workings of the innate immune system is slowly developing from the dark horse it once was into a frontrunner with the realization that the innate immune system is an integral part of immunity. By definition, the innate immune system is that part of the immune system that does not depend on previous exposures for optimal function. Its main functions are largely to act as a physical and chemical barrier, to recruit immune competent cells to the site of infection, to activate the adaptive immune system, and to activate the complement system. In this light, the innate immune system seems to buy time for the adaptive immune system to come to the rescue. However, it can also act as a fully accomplished eradicating defense system, avoiding the energy-consuming mobilization of the adaptive immune system. Research on the innate immune system over the years had focused on a number of larger topics. The first topic was the diverse role of (airway) epithelium in the innate defense. As the epithelium forms the outer layer of an organism, it is well-positioned to detect and respond to changes in the environment [1]. The second topic was the activity of different receptors by which cells can detect the outside environment, and the third was the action of (secreted) mediators that fight off potential threats. This review builds on the 2009 paper on the role of innate immunity in the pathogenesis of chronic rhinosinusitis (CRS) by Andrew Lane in this same journal [2••]. Starting from this point, in the first part of this review, we update and extend previous work, but in the second part, we focus on two main trends that could shape research, not only in the field of innate immunity, but also in the field of CRS and nasal polyposis (NP) [3, 4•, 5•].

The relationship between innate immunity and CRS has always been special. In cystic fibrosis, there is an absence of mucociliary clearance and a high prevalence of CRS. The presence of biofilms in CRS patients and the postulated roles for *Staphylococcus aureus*, viruses, and fungi in the pathogenesis of CRS over the years have all been based on a disrupted ability to fight off these microorganisms. Given that it is not clear to what extent the adaptive immune system is involved (aspecific activation of polyclonal T cells by superantigens, non–IgE-mediated antifungal response, local production of IgE in the absence of systemic allergy) in the complex inflammation of CRS [6, 7], it seems logical that the innate immune reactions are so carefully considered in the pathogenesis of CRS. We should, however, also not forget that CRS is not a single disease. Not only do some patients with CRS suffer from NP (while others do not), the differences in cellular composition between “Asian” (neutrophilic) and “Caucasian” (eosinophilic) polyps [8, 9] could even suggest that the strong focus we have placed on particular inflammatory cells in the pathogenesis of CRS has no merit. Moreover, CRS without NP may have different etiologies in different patients, which would make it difficult to find a common adaptive or innate immune response deficiency as the root course of the disease.

## Chronic Rhinosinusitis and the Outside Microbial World

Although CRS with NP inflammation is clearly associated with exaggerated production of T-helper type 2 (Th2) cytokines and pro-eosinophilic mediators [6, 7], the underlying cause remains unknown. Multiple groups have proposed that abnormal immune responses may accompany exposure to microorganisms and/or their products, including fungi, *S. aureus*, or bacterial biofilms [10–12]. This line of thought remained a main topic of research.

Given the postulated role of microorganisms in the pathogenesis of CRS or nasal polyposis, it seems reasonable to assume that trying to fight (specific) pathogens through medication would prove advantageous. However, unfortunately, there is at best modest success with this approach. Multiple placebo and controlled studies with antifungals showed no beneficial effect [13–15]. Despite the initial success [16], a recently published meta-analysis also showed that even placebo did better than the active arm [17]. The use of antibiotics or macrolides yields varying results with modest effect for roxithromycin [18] and for doxycycline [19], whereas an international study with low-dose azithromycin yielded no effect on multiple clinical parameters [20].

It is important to stress that the modest effect of antimicrobial treatment does not imply that innate immunity is not relevant for CRS or NP, as treating single classes of pathogens would still leave others (fungi vs bacteria vs virus); with microorganisms all around us, recolonization could be very rapid; and preventing growth of microorganisms or induction of their killing should still lead to the release of pathogen-associated ligands that could trigger the innate response. Interesting, although not related to the fight against pathogens but rather related to the Th2-like inflammation seen in CRS/NP, is the experimental use of an antibody directed against interleukin (IL)-5 [21, 22]. An international consortium is currently evaluating these promising initial clinical results further in a larger study.

### Chronic Rhinosinusitis and the Detection of Microorganisms

Toll-like receptors (TLRs) have been at the forefront of research into innate immunity. As such, they are the best described family of receptors by which cells can detect microorganisms in their environment [1]. Also in CRS-related research, there has been great interest in these receptors as triggers of innate immune reactions. However, recent findings show that we probably need to broaden our view. One of the first negative results comes from the field of TLR2, in which despite previous observations of a link between lower expression of TLR2 and difficult-to-treat CRS [23], none of the investigated polymorphisms in the TLR2 gene could be linked to the disease [24]. Interestingly, one possible explanation is that we should not consider one receptor or one trigger in isolation. Although the design of the study would have been better with the inclusion of more than one concentration of stimuli, Nonaka and coworkers [25] showed that in fibroblasts isolated from allergic NP patients, the sole addition of TLR2 to TLR5 agonists or IL-4 did not reveal any induction of the T-lymphocyte–associated mediator TARC (thymus and activation-regulated chemokine). However, when the TLR agonists were combined with IL-4, a strong induction was seen [25]. Although this observation could be related to the allergic phenotype and not to the CRS background of the patients in this study, this outcome does show that we should more carefully consider interaction between triggers, especially because in everyday life, we seldom encounter single triggers. The same group, however, also showed that nasal polyp fibroblasts are able to respond to the sole addition of TLR2 to TLR5 agonists via the production of macrophage inflammatory protein-3α [26].

The horizon is also broadened by the observation that an additional family of innate immunity receptors is also affected in CRS and NP. These NLR receptors (NOD-like receptors) are found within the cytoplasm of cells and can also be triggered by microbial-derived factors [1]. Three of these receptors (NOD-1, NOD-2, and NALP-3) were investigated in NP and were shown to be highly enriched in the epithelia of patients relative to healthy controls and shown to be downregulated by steroid treatment [27]. In addition to the NLR class of receptors, an as-yet-unknown receptor is held responsible for the detection and responsiveness of airway epithelia to chitin [28]. Chitin, a cell wall component not found in humans, is able to induce expression in air-interface–grown epithelial cells of an enzyme that is able to break down chitin (acidic mammalian chitinase [AMCase]), and of eotaxin-3, a mediator important in the functionality of eosinophils. Increased expression of AMCase and eotaxin-3 is also connected to treatment efficacy, in which polyps that respond well have higher levels of AMCase and eotaxin-3 than those that respond more poorly.

### Chronic Rhinosinusitis and the Battle Against Microorganisms

Lysozyme is a major component of nasal secretions and was always seen as an important antibacterial defense protein given its ability to break down bacterial cell wall. Woods and coworkers [29] now have shown that lysozyme can also play a role in the fight against fungi, as growth in cultures from multiple species, including *Aspergillus*, *Penicillium*, and *Candida,* is susceptible to lysozyme. This was also true for clinical isolates from CRS patients, showing that selection of fungi not responsive to lysozyme is not part of the pathological mechanism in CRS. Whether or not changes in lysozyme activity or expression could be involved in CRS remains to be explored. That changes in activity of antimicrobial agents could play a role is seen for lactoferrin. At both the gene expression level and the protein level, it was shown that lactoferrin was reduced in CRS patients compared with controls [30]. Small cationic peptide–like defensins and cathelicidins (LL37) make up the third important class of antimicrobial agents and have been described previously [2••], but a recent development was the realization that small lipids (cholesteryl esters) [31] that can be found in breast milk also can be found at increased levels in nasal secretions from CRS patients [32•]. Interestingly, expression of antimicrobial agents is highly variable among individuals suffering from disease [33] and may also depend on the colonization status of *S. aureus* in NP patients [34].

## A Global View on Innate Immunity and Chronic Rhinosinusitis

New lines of research seem to abandon the thought that a unique molecule, cell, or pathogen could be the single source of CRS in all patients. With the technical developments in genetics, transcriptomics, and proteomics, a more generalized approach is chosen. Although these approaches may suffer from their own restrictions [35], they can provide a broader picture. Perhaps even more importantly, as these methods collect a wide range of (molecular) data on single individuals, they may aid in defining molecular patterns that could point to subtypes of CRS and/or help explain the widely varying success rates of treatment among individuals suffering from CRS.

### Polymorphisms and Chronic Rhinosinusitis

Genome-wide association studies focus on differences at the genomic level between cases and controls. These differences are at the DNA sequence level, of which single nucleotide polymorphisms (SNPs) are the most commonly investigated and in which cases could represent individuals suffering from CRS or individuals with CRS who respond poorly to treatment. The rational is that SNPs could have consequences for the expression level of a given gene when the mutations are located in promoter or enhancer elements, or that these mutations could have consequences for the activity of the proteins encoded by the gene in question when the mutation is in the coding sequences. The elegant design used by the group of Desrosiers et al. has identified a growing list of SNPs in a growing list of genes that were shown to be different between a group of more than 200 CRS patients and close to 190 postcode-matched controls in Canada [24, 36–41]. The roles of the genes involved vary but seemed to center on the detection of potential pathogens [24], the detection of mediators [36, 37], and downstream signaling events [38–41]. Given the uniqueness of this group of cases and controls, it would be interesting and important to study the functional consequences of the discovered SNPs. Moreover, given that the genes belong to the larger group of signaling-related genes, it would be relevant to determine whether or not a correlation exists between the polymorphisms in the different genes. That the SNPs are all discovered in the same group of individuals does not necessarily imply that the polymorphisms in different genes coexist in the same individual. The potential relationship between these genes (and their SNPs) would provide a deeper understanding of the potential processes involved in the pathogenesis of CRS.

Some of the mutation studies focus on SNPs in genes that are directly linked to previous parts of this review, reflecting the absence of specific SNPs in TLR2 linked to CRS [24] or the presence of mutations linked to the functionality of the newly discovered innate lymphoid cell (ILC)2 (described in further detail subsequently) [36, 37]. One study focuses on SNPs within a member of the SERPIN family [41]. This family of proteins is named after the proteins’ ability to inhibit the protease activity of serine proteases. One of the important target molecules of SERPIN-A1 is the elastase produced by neutrophils. This protease can be released from neutrophils after exposure to bacterial superantigens or allergens and could therefore contribute to the ongoing inflammation in CRS. Indeed, one of the SNPs investigated was linked to a nearly sixfold increase in the risk of developing CRS. Whether this mutation had consequence for the activity or SERPIN-A1 or its secretion was not investigated. In contrast, no SNPs in glutathione S-transferase, a gene linked to redox state, could be linked to CRS [42].

### Transcriptomics, Proteomics, and Chronic Rhinosinusitis

The analysis of the transcriptome (the collection of mRNAs that are expressed) has also found its way in the study of the involvement of innate immunity in CRS. Several studies have specifically focused on innate immunity through the use of dedicated arrays (or polymerase chain reaction–based technologies) [43, 44], while others have investigated the complete transcriptome through microarray technologies [45–48]. The later studies can be seen to describe the potential consequences of the involvement of the innate immune system and other aspects of the disease that are not necessarily linked to innate immunity.

In a screen, among the expression of 125 TLR-related genes in nonatopic CRS patients and controls, 23 genes were shown to be differentially regulated [43]. Four genes were upregulated and 19 genes downregulated. Among the genes that were upregulated in disease were *TLR9* and *TLR10*, while among the genes that were downregulated, a substantial number belong to the JNK/p38 pathway. It is interesting to see that of the three main pathways [1] that are downstream of the TLRs (the nuclear factor-κB [NF-κB] pathway leading to activation of the NF-κB-family of transcription factors, the JNK/p38 pathway leading to the activation of the AP1 family of transcription factors, and the IRF pathway leading to the activation of type 1 interferons), one pathway is so strongly affected. The increased expression of TLR9 contrasts previous findings [23] that showed a reduced expression of TLR9. The reason for this difference is not clear, but it could be related to differences in the allergic status among the participants in these two studies—nonatopic in the study of Zhao and coworkers [43], (partly) atopic in the study of Lane and colleagues [23]. The question remains as to what are the functional consequences of the observed deregulation of genes, with on one hand the upregulation of the TLR receptor, and on the other hand a downregulation of part of the downstream signaling cascade. The complement system is the focus of the study by Schlosser et al. [44]. The complement system is important in the direct battle against pathogens that have penetrated the peripheral barriers. Overactivation of this system may result in uncontrolled inflammation, whereas inactivity may fail to eradicate potential threats. This is the first study to address the complement system in detail, although expression of a complement factor (C3) in CRS has been described previously [49]. A strongly increased expression of factor B (sixfold) and C3 (fourfold) was detected relative to controls in combination with a small increase in the terminal protein C5 (twofold) and no change in C7. Although most of the complement factors are produced in the liver, the increased expression of factor B and C3 not only suggests the involvement of the alternative pathway of activation, but also that these factors (in the absence of increased expression of the terminal proteins) could be locally produced by the airway epithelial cells [50]. The other microarray [45–48] and proteomic [51–55] studies, by their nature, have not investigated the involvement of the innate immunity directly but show (in addition to innate immunity–related genes and proteins) the global effects of the ongoing inflammation and its potential use as a predictor of treatment outcome or clinical classification.

## A New View on Innate Immunity and Chronic Rhinosinusitis

The immunology field recently witnessed an interesting development in the discovery of an extended family of ILCs that seem to play an important role in tissue remodeling and in the defense against microorganisms and helminthic parasites [56]. ILCs have been described previously, with natural killer (NK) cells and lymphoid tissue inducer (LTi) cells as the best known protagonists [57, 58]. New members of the ILC family share characteristics of NK cells and LTi cells in that they not only express NK markers such as NKp44 and NKp46, but also LTi markers such as CD127 (IL-7 receptor chain α) and the transcription factor RORγt [56]. Moreover, these cells are able to produce IL-17 or IL-22 and in this respect resemble some of the different subtypes of Th cells that play such an important role in the different branches of the adaptive immune system. The parallel with the adaptive immune system was further strengthened by the description of an ILC type that is able to produce the archetypal Th2 cytokines IL-5 and IL-13. Given their ability to produce IL-13, these cells have been named *nuocytes* (after the 13th letter of the Greek alphabet) or ILC2s (after their resemblance to Th2 cells) [59, 60]. They were originally described only in mice gut, but we have recently discovered that this cell type is highly enriched in nasal polyps of CRS patients [61••]. These cells produce IL-5 and IL-13 and respond to exposure to IL-25 and IL-33 produced by several cell types in nasal polyps, including macrophages, mast cells, and epithelial cells. This would tie in with the observation that epithelial cells isolated from nonresponsive polyps (return of visible polyps within 6 months after surgery despite postoperative use of local and oral steroids) showed higher basal mRNA levels of IL-33 than epithelial cells isolated from responsive polyps [25, 62]. Moreover, reports show that downstream of ILC2s, CRS with NP is associated with lower levels of expression of the epithelial IL-22 receptor compared with healthy individuals or CRS patients without NP [63], and that polymorphisms in the gene are correlated with disease severity [27]. Given the ability of nasal epithelial cells to respond to IL-13 and IL-22, such a potential should consider that IL-33 can also be produced by fibroblasts and that IL-33 receptors can also be found on mast cells, eosinophils, and Th2 lymphocytes [12]. Despite these obvious restrictions, the negative or positive feedback loop may contribute to the severity of the disease or its treatment. However, it is noteworthy that IL-33 mRNA levels are somewhat enhanced by the TLR agonist CpG in epithelial cells from treatment-resistant CRS, but not in epithelial cells from treatment-responsive CRS [64], which would again link CRS to aberrant antimicrobial immunity. Although the precise role of ILCs in general and ILC2s in particular in immune responses or the pathogenesis of CRS is still unknown, they could offer some explanation for the Th2/eosinophilic-like phenotype of NP without a prerequisite concomitant allergic response. Given the absence of RAG recombinase-dependent rearranged antigen receptors on ILCs, the immune responses mediated by these cells are most likely polyclonal in nature. However, exactly if and how ILC2s influence the evident aberrant adaptive immune responses seen in CRS inflammation remains unclear. Whether the discovery of ILCs heralds the end to the darkness that surrounds the pathogenesis of CRS or just adds another level of complexity surely will be the topic of interesting new lines of research.

## Conclusions

The marriage between CRS and/or NP remains strong, and there are no signs that the link will get weaker. There does seem to be a trend toward broader approaches that better consider that CRS is not a single disease and that no single pathogen, molecule, or cell is the cause of the disease. Although there will always be a need for dedicated research that would analyze the contribution of single pathogens, molecules, or cells to the pathogenesis of the disease or to its treatment success, techniques seem to have developed sufficiently to try to take an even broader view. Collecting data on the transcriptome, proteome, or metabolome (the collection of mRNA, proteins, or metabolic products) could assist in defining the disease in single individuals. Even when a single process would be the sole cause of CRS, the reason why this process is affected could be different in every patient. With CRS being a collection of different diseases in different patients, the need for a holistic view seems even more important. It might well be that our clinical options to categorize CRS are too limited by the sheer limitation of the number of clinical parameters we can use to describe the disease. In this respect, the discovery and description of molecular patterns may aid us in refining the clinical characterization of the disease and provide a better understanding as to why some treatments fail in certain individuals. CRS probably will always keep surprising and fascinating us. The discovery of a whole new class of innate lymphocytes that is so enriched in NP probably will not be the last of these surprises.
